# De-repression of CSP-1 activates adaptive responses to antifungal azoles

**DOI:** 10.1038/srep19447

**Published:** 2016-01-19

**Authors:** Xi Chen, Wei Xue, Jun Zhou, Zhenying Zhang, Shiping Wei, Xingyu Liu, Xianyun Sun, Wenzhao Wang, Shaojie Li

**Affiliations:** 1 State Key Laboratory of Mycology, Institute of Microbiology, Chinese Academy of Sciences, Beijing 100101, China; 2 University of Chinese Academy of Sciences, Beijing 100049, China; 3 Technical Centre of Beijing Cigarette Factory, Beijing 101121, China; 4 School of Marine Sciences, China University of Geosciences, Beijing 100083, China

## Abstract

Antifungal azoles are the major drugs that are used to treat fungal infections. This study found that in response to antifungal azole stress, *Neurospora crassa* could activate the transcriptional responses of many genes and increase azole resistance by reducing the level of conidial separation 1 (CSP-1), a global transcription repressor, at azole-responsive genes. The expression of *csp-1* was directly activated by the transcription factors WC-1 and WC-2. Upon ketoconazole (KTC) stress, the transcript levels of *wc-1* and *wc-2* were not changed, but *csp-1* transcription rapidly declined. A chromatin immunoprecipitation-quantitative polymerase chain reaction analysis revealed a rapid reduction in the WC-2 enrichment at the *csp-1* promoter upon KTC treatment, which might be responsible for the KTC-induced *csp-1* downregulation. Deletion of *csp-1* increased resistance to KTC and voriconazole, while *csp-1* overexpression increased KTC susceptibility. CSP-1 transcriptionally repressed a number of azole-responsive genes, including genes encoding the azole target ERG11, the azole efflux pump CDR4, and the sterol C-22 desaturase ERG5. Deletion of *csp-1* also reduced the KTC-induced accumulation of ergosterol intermediates, eburicol, and 14α-methyl-3,6-diol. CSP-1 orthologs are widely present in filamentous fungi, and an *Aspergillus fumigatus* mutant in which the *csp-1* was deleted was resistant to itraconazole.

Antifungal azoles, including imidazoles and triazoles, are the major drugs used to treat fungal infections. Some azoles are also applied as pesticides. Azoles disrupt ergosterol biosynthesis by inhibiting the 14α-demethylase Erg11p (also referred to as Cyp51p). In addition to blocking ergosterol synthesis, azoles cause the accumulation of a toxic sterol, 14α-methyl-3,6-diol^1^.This toxic sterol exerts severe membrane stresses on the cell^2^.

Fungi are able to adapt to azole stress by altering the expression of many genes. Overexpression of some azole-responsive genes, such as the azole target gene *ERG11* and azole pump-encoding genes, has been demonstrated to increase azole resistance in many fungi^3^,^4^,^5^,^6^,^7^,^8^. In previous studies of fungal adaptation and resistance to antifungal azoles, the majority of the efforts were focused on genes that were upregulated during azole stress. However, for most of the genes that were downregulated under azole stress, the effects of their downregulation were not studied in depth. Thus, these downregulated genes, especially those with regulatory functions, are potential regulators of azole adaptation.

Conidial separation 1 (CSP-1) is a global transcription repressor with a C2H2 zinc finger DNA-binding domain. CSP-1 is critical for conidial separation in *Neurospora crassa*^9^. CSP-1 orthologs are widely present in fungi. Its orthologs, Nrg1p and Nrg2p, in *Saccharomyces cerevisiae* have been well studied^10^. Nrg1p recruits the Tup1–Ssn6 complex to repress the transcription of its target genes^11^. Similarly, CSP-1 also physically interacts with RCO-1 (a Tup1p homolog) and RCM-1 (an Ssn6p homolog) in *N. crassa*^10^. In addition, Nrg1p and CSP-1 have the common consensus binding motif CCCT^10^,^11^,^12^. In *S. cerevisiae*, Nrg1p regulates glucose metabolism and deletion of either *NRG1* or *NRG2* enhances the resistance of cells to salt and oxidative stress, and decreases tolerance to freezing^13^. In *Candida albicans*, Nrg1p is critical for invasive growth and the morphological switch between yeast and hyphae^14^. Our previously published digital gene expression (DGE) data showed that *csp-1* was downregulated by ketoconazole (KTC) stress^15^. In this study, we demonstrated that downregulation of *csp-1* promoted transcriptional responses by several genes to KTC and conferred resistance to the drug. *csp-1* transcription is directly activated by the white-collar complex (WCC)^16^, which is composed of two transcription factors, WC-1 and WC-2^17^,^18^. The WCC is the core regulator of circadian rhythm and light responses^17^. In this study, we demonstrated that KTC stress rapidly reduced WC-2 enrichment at the *csp-1* promoter, which sheds light on the mechanism of *csp-1* downregulation during KTC stress. We also showed that deleting either *wc-1* or *wc-2* increased KTC resistance.

## Results

### *csp-1* expression is downregulated under KTC stress

Our previous DGE data showed that the azole target-encoding gene *erg11* (NCU02624), the sterol C-22 desaturase-encoding gene *erg5* (NCU05278), and the azole pump-encoding gene *cdr4* (NCU05591) showed dramatic transcriptional increases upon KTC treatment in a wild-type (WT) *N. crassa* strain^15^. However, the expression of the gene (NCU02713) encoding the zinc finger transcription factor CSP-1, which was previously identified as a regulator of conidial separation in *N. crass*^9^, was downregulated under KTC stress^15^. The transcriptional downregulation of *csp-1* under KTC stress was confirmed by quantitative real-time polymerase chain reaction (qRT-PCR). After 24 h of KTC treatment (2.5 μg/mL), the level of the *csp-1* transcript decreased by 77 ± 3% (p = 0.0005, n = 3).

### Deletion of *csp-1* increases azole resistance

**Figure 1:**
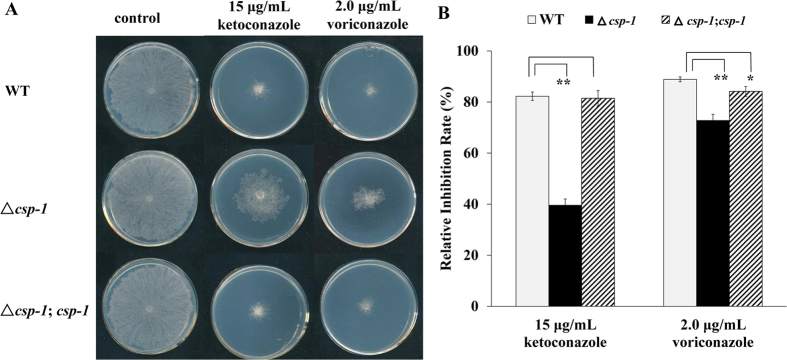
Deletion of *csp-1* reduces azole sensitivity. (**A**) Azole sensitivity test. The mycelial plugs of wild-type, ∆*csp-1*, and ∆*csp-1*, *csp-1* strains were inoculated onto plates, with or without azoles, and incubated at 28 °C in the dark. Images of colonies were captured after 24 h. (**B**) Relative growth inhibition rates. Relative growth inhibition rates were calculated based on colony diameters. Values of three replicates were used for a statistical analysis. Means of the inhibition rates are shown, and standard deviations are marked with bars. Values with extremely significant (P < 0.01) and significant differences (0.01 < P < 0.05) are marked with** and *, respectively.

To understand the significance of *csp-1* downregulation in azole adaptation, a *csp-1* deletion mutant (Fungal Genetics Stock Center (FGSC) #11348) was subjected to a drug sensitivity test. Because conidia in the ∆*csp-1* mutant could not be separated, the minimum inhibitory concentrations of antifungal azoles could not be measured. To test its drug susceptibility, we inoculated mycelial mats of the WT and ∆*csp-1* strains onto solid Vogel’s plates, with or without azoles, and compared their growth. On the drug-free plates, the growth rate of the ∆*csp-1* mutant was similar to that of the WT strain. On plates supplemented with KTC (15 μg/mL) or voriconazole (2.0 μg/mL), the colony growth of both strains was inhibited. The inhibition rates of the WT strain by KTC and voriconazole were 82.26 ± 1.65 and 88.89 ± 0.97%, respectively, while the inhibition rates of the ∆*csp-1* strain were only 39.57 ± 2.47 and 72.76 ± 2.42%, respectively (Fig. 1). A statistical analysis using a *t*-test indicated that the inhibition rates for both azoles significantly differed between the WT and ∆*csp-1* strains (p_ket_ = 0.00005, n = 3; p_vori_ = 0.0030, n = 3), suggesting that the transcriptional downregulation of *csp-1* increases azole resistance. Complementation of the ∆*csp-1* mutant resulted in a WT level of azole susceptibility (Fig. 1). The inhibition rates of the complemented strain (∆*csp-1*, *csp-1*) by KTC and voriconazole were 81.47 ± 3.03 and 84.15 ± 1.95% (p_ket_ = 0.7195, n = 3; p_vori_ = 0.0337, n = 3), respectively, which were not significantly different from that of the WT strain. For other stresses, including the antifungal drug benomyl (0.5 μg/mL), osmotic stress (1 M NaCl), and high temperature (42 °C), the *csp-1* deletion mutant showed WT sensitivities (Supplemental Figure S1), suggesting the functional specificity of CSP-1 in the azole response.

### Overexpression of *csp-1* increases KTC sensitivity

**Figure 2:**
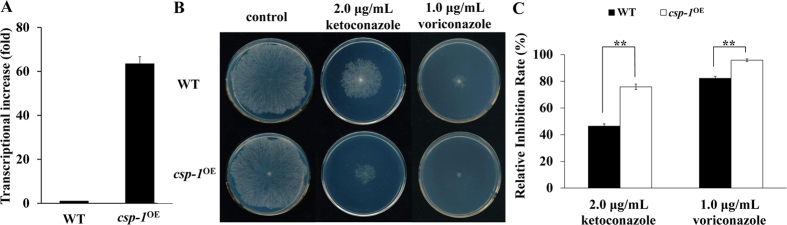
Overexpression of *csp-1* increases azole sensitivity. (**A**) Transcripts of *csp-1* detected by quantitative real-time polymerase chain reaction. The transcriptional increase of *csp-1* was calculated relative to the transcript level in the WT strain. Values shown are means of three replicates. Standard deviations are indicated with error bars. (**B**) Azole sensitivity test. Conidial suspensions of the WT and *csp-1* overexpression strain (*csp-1*^OE^) were inoculated onto solid medium, with or without azoles, and incubated at 28 °C in the dark. Images were captured at 24 h for the control and at 48 h for the azole-treated plates (at 24 h, none of the tested strains formed colonies on the plates containing azoles). (**C**) Relative inhibition rates. The calculation followed the method described in Fig. 1B. p_ket_ = 0.00005, n = 3; p_vori_ = 0.0005, n = 3.

To further confirm the role of the downregulation of *csp-1* expression in azole adaptation, a *csp-1* overexpression strain (*csp-1*^OE^) was generated, in which the expression of *csp-1* was driven by the *cfp* promoter^19^. A qRT-PCR analysis showed that the *csp-1* transcript level in the *csp-1*^OE^ strain increased significantly compared with that of the WT strain (Fig. 2A). On normal Vogel’s solid medium, the growth rate of the *csp-1*^OE^ strain was similar to that of the WT strain. However, on solid medium containing KTC (2.0 μg/mL) or voriconazole (1.0 μg/mL), the *csp-1*^OE^ strain grew significantly slower than the WT strain (Fig. 2B), indicating that the overexpression of *csp-1* increased KTC sensitivity. These results, together with the azole-resistant phenotype of the ∆*csp-1* strain, strongly demonstrate that CSP-1 plays a negative role in azole resistance.

### Deletion of either *wc-1* or *wc-2* causes KTC resistance

**Figure 3:**
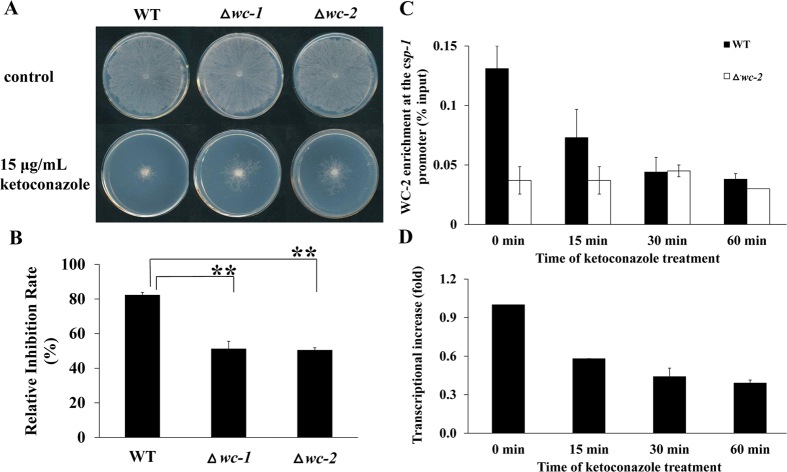
The WCC regulates ketoconazole (KTC) sensitivity and mediates the adaptive response of *csp-1* to KTC. (**A**) KTC sensitivity test of the ∆*wc-1* and ∆*wc-2* mutants. Mycelial plugs were inoculated onto plates, with or without KTC, and incubated at 28 °C in the dark. Images of colonies were captured after 24 h. (**B**) Relative inhibition rates. The calculation followed the method described in Fig. 1B. p_*wc-1*_ = 0.0029, n = 3; p_*wc-2*_ = 0.00002, n = 3. (**C**) ChIP-qPCR analysis of the WC-2 enrichment at the *csp-1* promoter. The *csp-1* promoter region associated with WC-2 was precipitated by an anti-WC-2 antibody and quantitatively measured by quantitative real-time polymerase chain reaction (qRT-PCR). The ratio of the qRT-PCR results between the chromatin immunoprecipitation sample and its input sample was used to indicate the level of WC-2 enrichment at the *csp-1* promoter. Means of the results from three independent experiments are shown. (**D**) *csp-1* transcripts after KTC treatment were detected by qRT-PCR. Values shown are the means of three replicates. Standard deviations are indicated with error bars.

The transcription of *csp-1* is directly activated by the WCC^16^, which is composed of two transcription factors, WC-1 and WC-2^17^. The WCC is the core regulator of circadian rhythm and light responses^17^. To find a connection between CSP-1 and the WCC in azole adaptation, we analyzed the KTC sensitivity of single *wc-1* and *wc-2* deletion mutants. The results showed that deleting either *wc-1* or *wc-2* significantly increased KTC resistance (Fig. 3A,B), indicating that WC-1 and WC-2 are also involved in azole adaptation.

### KTC induces a rapid reduction of WC-2 enrichment at the *csp-1* promoter

*csp-1* transcription is directly regulated by the WCC, and the binding locus of WC-2 at the promoter of *csp-1* has been reported^16^. Thus, the downregulation of *csp-1* expression might be caused by a reduction of WC-2 enrichment at the *csp-1* promoter under KTC stress. To analyze the kinetics of WC-2 enrichment at the *csp-1* promoter during KTC stress, the DNA associated with WC-2 was isolated by chromatin immunoprecipitation (ChIP) from the WT and ∆*wc-2* (the negative control) strains, and quantitatively measured by qPCR. The ratio of the qPCR results between the WC-bound DNA sample and the total input was used to indicate the WC-2 enrichment at the *csp-1* promoter. As shown in Fig. 3C, after 15, 30, and 60 min of KTC treatment, WC-2 enrichment at the *csp-1* promoter was reduced by 44.27 ± 2.37, 66.41 ± 1.25, and 70.99 ± 0.47%, respectively, compared with that of a non-treated sample (Fig. 3C). In contrast, WC-2 enrichment at the *csp-1* promoter region was relatively stable over time in the *wc-2* deletion mutant. These results indicate that WC-2 enrichment at the *csp-1* promoter region is reduced rapidly under KTC stress. Accordingly, *csp-1* transcript levels were reduced by 42.00 ± 0.14, 56.00 ± 6.68, and 61.00 ± 2.39% after 15, 30, and 60 min of KTC treatment, respectively (Fig. 3D). Thus, the reduction of WC-2 enrichment at the *csp-1* promoter correlated with the downregulation of *csp-1* transcription.

### CSP-1 regulates KTC-responsive genes

CSP-1 functions as a transcriptional repressor. A previous ChIP-sequencing analysis identified 920 genes that are directly regulated by CSP-1 in *N. crassa*^10^. By searching the list of our previously published KTC-responsive genes of *N. crassa*^15^, we found that 25.5% (235) of these 920 genes belong to the KTC-responsive genes (Supplemental Table S1), indicating that CSP-1 is a global regulator of transcriptional responses to KTC stress (Supplemental Table S1).

### Deletion of *csp-1* enhances transcriptional responses to KTC

From the above-mentioned 235 KTC-responsive genes, genes important for azole resistance were selected, and qRT-PCR was used to confirm the roles of CSP-1 in their transcriptional responses.

**Figure 4:**
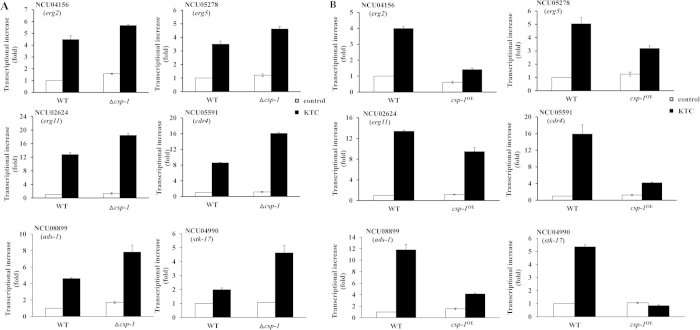
CSP-1 regulates transcriptional responses to ketoconazole(KTC) stress. (**A**) Effects of *csp-1* deletion (∆*csp-1*) on KTC transcriptional responses. (**B**) Effects of *csp-1* overexpression (*csp-1*^OE^) on KTC transcriptional responses. Transcript levels were measured by quantitative real-time polymerase chain reaction. Values shown are the means of three replicates. Standard deviations are indicated with error bars. *t*-test results are shown in Tables S2 and S3.

Azoles inhibit ergosterol biosynthesis, and several genes involved in ergosterol biosynthesis, including *erg2* (NCU04156), *erg5* (NCU05278), and *erg11* (NCU02624), are regulated by CSP-1, and their transcription increases in response to KTC stress^10^,^15^. A qRT-PCR analysis showed that in liquid Vogel’s medium without KTC, only *erg2* in had a significantly higher transcript level in the ∆*csp-1* strain than in the WT strain (p_*erg2*_ = 0.0017, n = 3). The transcript levels of *erg5* and *erg11* did not differ significantly between the mutant and WT strains (p_*erg5*_ = 0.0663, n = 3; p_*erg11*_ = 0.1747, n = 3) (Fig. 4A; Supplemental Table S2). Upon KTC treatment (2.5 μg/mL), the transcript levels of *erg2*, *erg5*, and *erg11* increased dramatically in the WT and mutant strains. However, the transcript levels of these genes were higher in the mutant than in the WT strain (Fig. 4A). A statistical analysis showed that the transcript levels of these genes differed significantly between the *csp-1* mutant and WT strain (p_*erg2*_  = 0.0239, n = 3; p_*erg5*_ = 0.0032, n = 3; p_*erg11*_ = 0.0004, n = 3) (Supplemental Table S2).

In addition to these ergosterol biosynthesis genes, we also chose two CSP-1-regulated genes, NCU04990 and NCU08899, from the list of KTC-responsive genes. The NCU04990 and NCU08899 single gene deletion mutants were hypersensitive to KTC (data not shown). NCU04990 encodes a homolog of the kinase STK-17, and NCU08899 encodes a novel transcription factor that was named ADS-1 (*a*ntifungal *d*rug *sensitive-1*) in this study. Under KTC stress, the transcript levels of NCU04990 and NCU08899 increased by 1.99 ± 0.13- and 4.62 ± 0.09-fold, respectively, in the WT strain (Fig. 4A). In the *csp-1* deletion mutant, the transcript levels of NCU08899 and NCU04990 increased by 4.63 ± 0.52- and 7.82 ± 0.85-fold, respectively, under KTC stress (Fig. 4A).

CDR4, an ortholog of *C. albicans* Cdr1p and *S. cerevisiae* Pdr5p, is the key azole efflux pump in *N. crassa*^20^. Although *cdr4* was not reported to be a direct target of CSP-1^10^, we found that the antisense chain of its 5′ upstream region contains two CSP-1 binding sites (the CCCT motif) in the sequence TCCCTGAGTCCCAGGT (from −141 to −157 bp). Thus, we also analyzed the effects of *csp-1* deletion on *cdr4* expression. In the absence of KTC, *cdr4* transcript levels did not differ significantly between the WT and mutant strains (p_*cdr4*_ = 0.1331, n = 3) (Supplemental Table S2). Upon KTC treatment, *cdr4* transcript levels dramatically increased in the WT and mutant strains (Fig. 4A). However, the *cdr4* transcript level was higher in the mutant than in the WT strain (Fig. 4A). The difference in *cdr4* transcript levels between the mutant and WT strain was statistically significant (p_*cdr4*_ = 0.00002, n = 3) (Supplemental Table S2).

Because deleting *csp-1* increased the transcripts of the above-mentioned azole-responsive genes under KTC stress, a reduction of *csp-1* expression can reduce its repressive effects on these azole-responsive genes.

### Overexpression of *csp-1* compromises transcriptional responses to KTC

The effects of *csp-1* overexpression on the transcriptional responses of *erg2*, *erg5*, *erg11*, *ads-1*, *stk-17*, and *cdr4* to KTC stress were analyzed by qRT-PCR. In liquid Vogel’s medium without KTC, *csp-1* overexpression reduced the transcript levels of *erg2, ads-1*, and *erg11*, but not those of *erg5*, *stk-17*, and *cdr4* (p_*erg2*_ = 0.0001, n = 3; p_*erg5*_ = 0.0941, n = 3; p_*erg11*_ = 0.0430, n = 3; p_*ads-1*_ = 0.0231, n = 3; p_*stk-17*_ *= *0.3727, n = 3; p_*cdr4*_ = 0.1012, n = 3) (Fig. 4B; Supplemental Table S3). KTC treatment (2.5 μg/mL) caused much greater differences in the transcript levels of these genes between the WT and *csp-1* overexpression strains, although the transcript levels of *erg2*, *erg5*, *erg11*, *ads-1*, *stk-17*, and *cdr4* increased in the WT and *csp-1* overexpression strains. After 24 h of KTC treatment, the transcript levels of these genes were significantly lower in the *csp-1* overexpression strain than in the WT strain (p_*erg2*_ = 0.0000, n = 3; p_*erg5*_ = 0.0103, n = 3; p_*erg11*_ = 0.0079, n = 3; p_*ads-1*_ = 0.0044, n = 3; p_*stk-17*_ = 0.0001, n = 3; p_*cdr4*_ = 0.0112, n = 3) (Fig. 4B; Supplemental Table S3). Thus, *csp-1* overexpression compromises the transcriptional responses by these genes in response to KTC, further indicating that the downregulation of *csp-1* expression promotes transcriptional responses to KTC stress.

### Deletion of *csp-1* reduces the accumulation of ergosterol intermediates during KTC stress

**Figure 5:**
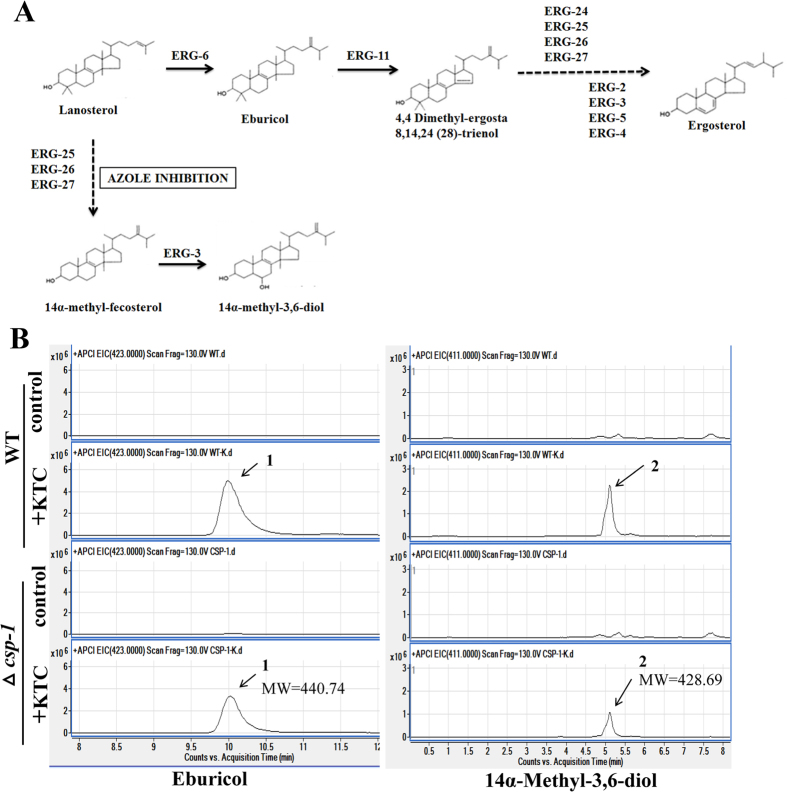
High-performance liquid chromatography-mass spectrometry (HPLC-MS) chromatogram of sterol extracts. (**A**) A schematic representation of the ergosterol biosynthetic pathway. (**B**) HPLC-MS chromatograms for eburicol and 14α-methyl-3,6-diol in the wild type and ∆*csp-1* strains with/without ketoconazole treatment. The derived sterols were identified by their molecular weights.

The above results indicate that the *csp-1* transcript level has a significant impact on the expression of three ergosterol biosynthesis genes under KTC stress. To determine the role of CSP-1 in ergosterol biosynthesis under KTC stress, we compared the sterol compositions between the WT and ∆*csp-1* strains by high-performance liquid chromatography-mass spectrometry (HPLC-MS). Under KTC stress, eburicol (a substrate of ERG11) and 14α-methyl-3,6-diol (a toxic14α-methylated sterol), two intermediates in the ergosterol biosynthesis pathway, accumulated^21^,^22^. As shown in Fig. 5, in the absence of KTC, eburicol and 14α-methyl-3,6-diol were almost undetectable in the WT and ∆*csp-1* strains. After 24 h of KTC treatment (2.5 μg/mL), eburicol and 14α-methyl-3,6-diol accumulated in the WT and mutant strains. However, the WT strain had higher levels of these two ergosterol intermediates than the mutant (Fig. 5). Based on the results from two independent experiments, the level of eburicol in the KTC-treated ∆*csp-1* strain was about 67.05 ± 8.24% of that in the KTC-treated WT strain, and the level of 14α-methyl-3,6-diol in the KTC-treated ∆*csp-1* strain was 54.55 ± 15.61% of that in the KTC-treated WT strain.

These data indicate that CSP-1 has a protective role against the accumulation of these ergosterol intermediates under KTC stress. Thus, downregulation of *csp-1* expression enables cells to maintain a normal sterol composition.

### Deletion of Af*csp-1* increases itraconazole resistance in *Aspergillus fumigatus*

**Figure 6:**
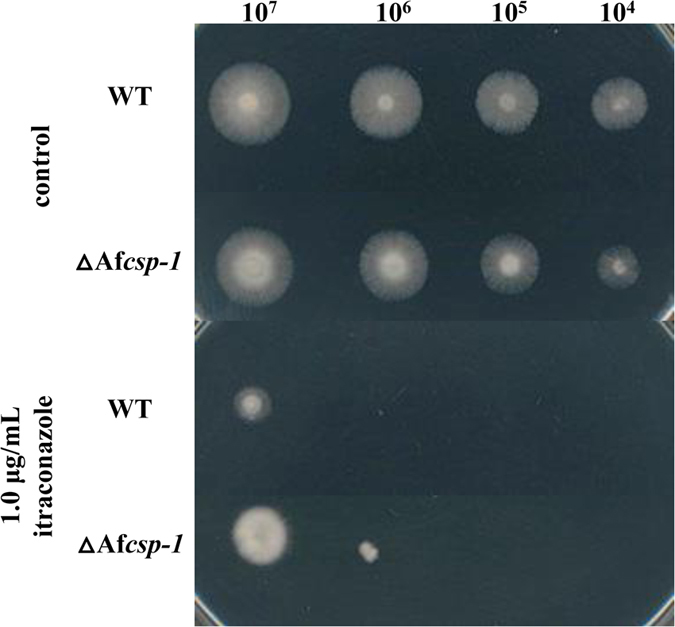
Drug sensitivity test of the Af*csp-1* deletion strain. Conidial suspensions at different concentrations (from 1 × 10^4^ to 1 × 10^7^ conidia/mL) of the wild type and Af*csp-1* (∆Af*csp-1*) deletion strains were inoculated onto complete medium, with or without itraconazole, and incubated at 37 °C in the dark. Images of the strains were captured after 24 h for the non-drug treated plate and 72 h for the itraconazole-treated plate.

CSP-1 orthologs are widely present in fungi. To investigate whether CSP-1 orthologs have similar functions in other filamentous fungi, we chose the opportunistic pathogen *Aspergillus fumigatus* as a test species. The CSP-1 ortholog in *A. fumigatus* is Afu1g10230, named AfCSP-1 in this study. AfCSP-1 shares 48% sequence similarity with CSP-1. An Af*csp-1* deletion mutant was generated by protoplast transformation. On an agar plate without drugs, the mutant displayed a WT growth rate and normal conidiation (Fig. 6). On an agar plate containing itraconazole (1 μg/mL), the deletion mutant showed greater itraconazole tolerance than the WT strain (Fig. 6), suggesting that CSP-1 orthologs are functionally conserved among fungi in azole adaptation.

## Discussion

Gene transcription is regulated by both positive and negative regulators. To investigate azole responses and resistance in fungi, most studies have tried to understand how azole-responsive genes are activated during antifungal azole stresses, which led to the identification of some positive regulators of azole responses and resistance^23^−^26^. To date, only two negative regulators of azole responses, Fcr1p in *C. albicans* and Stb5p in *Candida glabrata*, were identified^27^,^28^. Although the downregulation of *FCR1* expression was also observed during fluconazole stress, the mechanism that explains how *FCR1* expression is downregulated during azole stress is still unknown. In addition, their homologs were not found in filamentous fungi, suggesting that filamentous fungi might have unknown negative regulators of azole responses. Using *N. crassa* as a model, this study identified the first group of negative regulators of azole responses in filamentous fungi. Although the functions of CSP-1, WC-1, and WC-2 have been intensively investigated, their roles in azole adaptation and resistance were not previously known. The results of this study suggest that in response to antifungal azole stress, fungi could activate transcriptional responses and increase resistance by releasing the transcriptional repression of CSP-1 on azole-responsive genes.

**Figure 7:**
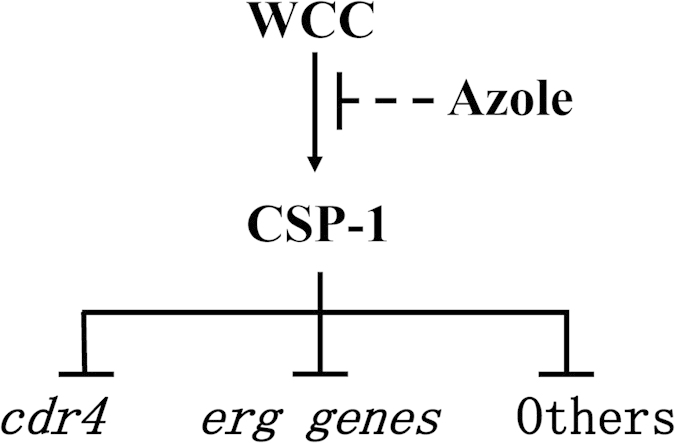
A schematic model of the regulation of azole responses by the WCC and CSP-1.

A number of studies have demonstrated that WC-1 and WC-2 function by forming the WCC^17^,^29^. The similar azole-resistance phenotypes of their single gene deletion mutants suggest that the formation of the WCC is likely required for their function under azole stress. Although we did not determine the enrichment of WC-1 at the *csp-1* promoter, the results of the WC-2 enrichment at the *csp-1* promoter should theoretically reflect the kinetics of the WCC at the *csp-1* promoter. Based on the results of this study and our knowledge of WC-1, WC-2, and CSP-1, the roles of CSP-1 and the WCC in the azole response can be explained by the model shown in Fig. 7. In the absence of azoles, the WCC strongly binds to the *csp-1* promoter, and *csp-1* is actively expressed. Consequently, the transcription of many azole-responsive genes is repressed by CSP-1. Under azole stress, WCC enrichment at the *csp-1* promoter is weakened by unknown mechanisms and, thus, *csp-1* expression is reduced. Therefore, the transcriptional repression of azole-responsive genes by CSP-1 is compromised. As a result, the transcript levels of azole-responsive genes increase under azole stresses. Based on the functions of CSP-1-regulated, azole-responsive genes, we hypothesize that CSP-1 exerts its impacts on azole response and adaptation by the following mechanisms.

First, CSP-1 regulates the adaptation of lipid metabolism to azole stress. Antifungal azoles disrupt ergosterol biosynthesis. The accumulation of the ergosterol intermediates eburicol and 14α-methyl–3,6-diol under KTC stress indicates that antifungal azoles have dramatic affects on sterol composition. Many genes involved in ergosterol biosynthesis have transcriptional responses to azole stress^10^,^30^,^31^. The transcriptional responses of *erg11* and *erg5* confer azole resistance^8^,^21^. These adaptive transcriptional responses might be useful to maintain the relative stability of the sterol composition. Deletion of *csp-1* enhanced the responses by genes involved in ergosterol biosynthesis to KTC stress, and reduced the accumulation of the ergosterol intermediates eburicol and 14α-methyl-3,6-diol. Thus, the transcriptional regulation of genes involved in ergosterol biosynthesis by CSP-1 under azole stress is beneficial for the stability of the sterol composition. In addition to ergosterol biosynthesis, a number of genes involved in the metabolism of other membrane lipid components also exhibit transcriptional responses to antifungal azoles^26^,^32^. In *S. cerevisiae*, deleting the genes *SKN1*, *IPT1*, or *SUR1*, which are involved in sphingolipid biosynthesis, resulted in miconazole resistance^30^, suggesting that some alterations in the metabolism of other lipid components could also increase azole resistance. Actually, CSP-1-regulated lipid metabolic genes are not limited to ergosterol biosynthesis. A total of 59 genes that are involved in lipid metabolism have CSP-1 binding sites at their promoters^9^. Among these genes, 26 displayed transcriptional responses to KTC stress (Supplemental Table S1). Thus, in addition to ergosterol, other lipid-metabolizing genes also exhibit adaptive alterations under azole stresses. Downregulation of *csp-1* expression will alter their expression under azole stress and, consequently, contribute to the adaptation of lipid metabolism to azole stresses.

Second, CSP-1 regulates the adaptive response of *cdr4*, the gene that encodes the major azole efflux pump, to azole. Azole efflux pumps, which pump azoles out of cells, are the key contributors in azole adaptation and resistance^3^,^4^,^5^. Among the four Pdr5p homologs in *N. crassa*, only CDR4 (NCU05591) has been shown to contribute to azole resistance^20^. Although a previous study did not report whether *cdr4* was a CSP-1 target gene^10^, we discovered CSP-1 binding sites (the CCCT motif) at its promoter region. In addition, CSP-1-regulated genes are also involved in other functions,^10^ and many of these genes transcriptionally responded to KTC stress (Supplemental Table S1), suggesting that CSP-1 might use this mechanism to regulate azole responses and resistance.

WCC, the core regulator of light responses and circadian rhythm in *N. crassa*, is responsible for the downregulation of *csp-1* expression during azole stress. WC-1 and WC-2 contain the multifunctional domain PAS (PER-ARNT-SIM)^33^. In animals, PAS domains are mainly involved in protein-protein interactions, and they are widely distributed in proteins that sense environmental changes^34^,^35^. Given this, we hypothesize that the WCC might function as a stress sensor or a key regulator that increases/decreases the transcription of relevant genes under azole stress. The WCC-sensed stress might result from the drug itself or from abnormal sterol metabolites. Then, the stress signal might modify the WCC and reduce its binding activity, thereby leading to the further downregulation of *csp-1* expression. As a global transcriptional repressor, the downregulation of CSP-1 expression will, in turn, promote the expression of its suppressed genes, including genes involved in azole adaptation and resistance (Fig. 7). Although this study added three new components to the regulatory mechanisms of azole responses and resistance, and partially revealed their functions in azole stress, many questions related to their detailed roles in azole stress are still unclear and should be addressed in future studies. Key questions include: 1) What are the direct signals that alter the binding activity of the WCC at the *csp-1* promoter? 2) How is the transcriptional activity of the WCC rapidly altered during azole stress? 3) Because the WCC is essential for blue light responses and circadian rhythm in *N. crassa*, is azole susceptibility influenced by light or circadian rhythm?

## Methods

### Strains and culture conditions

All of the single gene deletion mutants of *N. crassa* used in this study were purchased from the FGSC (Kansas City, MO, USA) (Table 1). The *csp-1* overexpression strain was created in this study. The primary medium used in this study included a solid slant medium (1 × Vogel’s salts, 2% sucrose, and 1.5% agar), a solid plate medium (1 × Vogel’s salts, 2% glucose, and 1.5% agar), and a liquid medium (1 × Vogel’s salts, and 2% glucose).

The *A. fumigatus* WT strain YJ-407 and the *pyrG*-defective strain CEA17 were obtained from Cheng Jin’s laboratory^36^. Strains were cultured at 37 °C on complete medium (10 g/L glucose, 2 g/L peptone, 1 g/L yeast extract, 1.5 g/L casein hydrate, and 2% salt solution), or minimal medium (10 g/L glucose and 2% salt solution) containing 0.5 mM sodium glutamate as a nitrogen source. Uridine (5 mM) and uracil (10 mM) were added when growing the CEA17 strain^36^.

### Drug sensitivity test

Azoles were dissolved in dimethyl sulfoxide and then aseptically added to the medium as needed. For the strain with a defect in conidial separation (the *csp-1* deletion mutant), mycelium plugs were used for the drug sensitivity test. Specifically, strains were inoculated on normal, solid Vogel’s medium. After 24 h of incubation at 28 °C in the dark, colonies formed, and then mycelial plugs (Φ = 5.5 mm) were taken from 0.5 cm behind the colony front and transferred to new Petri dishes, with or without azoles. The mycelium plugs of the WT strain were used as controls. For the strain with normal conidiation, 2 μL of a conidial suspension (2 × 10^6^ conidia/mL) was inoculated onto the center of agar plates (Φ = 9 cm) with or without azoles.

### RNA extraction and qRT-PCR analysis

Mycelial pieces (Φ = 1 cm) were inoculated into flasks containing 100 mL of Vogel’s liquid medium^15^. The cultures were incubated at 28 °C with shaking at 180 rpm for 12 h in the dark, and then KTC was added to a final concentration of 2.5 μg/mL. After 24 h, mycelia were collected for RNA extraction.

RNA extraction and qRT-PCR analysis were performed following previously described methods^15^. Each cDNA sample was analyzed in triplicate, and the average threshold cycle was calculated. The expression of genes was normalized to the β-tubulin expression level. Relative expression levels were calculated using the 2^−∆∆Ct^ method^37^. Gene-specific primers are shown in Table 2.

### Generation of the *csp-1* overexpression strain

The minimal *cfp* promoter was used to overexpress *csp-1*^19^. The *cfp* promoter (888 bp) was amplified from the *N. crassa* WT genome by PCR using primers cfp-F (5′-CGACCTCAAACCTCAACAAAC-3′) and cfp-R (5′-TTTGCCCTCGTGACTAAGAAGACCCTTCTTGG-3′). The *csp-1* coding region, with a 5 × Myc-6 × His tag, was amplified from the pQa5myc6his-*csp-1* vector (constructed by inserting the *csp-1* coding sequence into pQa5myc6his) by PCR using the primers hismyccsp1-F (5′-GAGAGAGCATCGGATCTGATATCATCGATTTAAAGC-3′) and hismyccsp1-R (5′-TTTGCCCTCGTGACTAAGAAGACCCTTCTTGG-3′). The *trpC* terminator (997 bp) was amplified from the pCSN43 (FGSC) vector using primers trpc-F1 (5′-TTCTTAGTCACGAGGGCAAAGGAATAGAGTAG-3′) and trpc-R1 (5′-AAGCAGCCCAGTAGTAGGTTGA-3′).

These PCR fragments were joined by fusion PCR and then inserted into the pCSN43 vector that was digested with EcoRV. The resulting construct, p*myc-his-csp-1*, was transformed into strain FGSC #4200 (the WT strain) by electroporation^38^, and transformants were screened by hygromycin resistance and verified by PCR using primers cfp-F and trpc-R1.

### Complementation of the ∆*csp-1* and ∆*wc-2* mutants

*csp-1*- and *wc-2*-containing fragments were obtained by PCR using primer pairs Comcsp-1-F (5′-GAGGAGATGCTGACTCGGTT-3′) and Comcsp-1-R (5′-GCTCTAGAGAAGGTTGGAAGGATGGAAA-3′), and Comwc-2-F (5′-CGGTGACAAACCCGAAGTAG-3′) and Comwc-2-R (5′-CCGCTCGAGGGTCGCCGTAGTAATCAGAAG-3′), respectively. The *csp-1* PCR product was digested with XbaI and then inserted into pCB1532 (FGSC) at the XbaI site to form the complementation plasmid pCB1532-*csp-1*. To obtain the complementation plasmid pCB1532-*wc-2* for the ∆*wc-2* mutant, the *wc-2* PCR product was digested by Xhol and then inserted into pCB1532 (FGSC). Transformations were performed using a previously reported method^39^. Transformants were screened on Vogel’s medium containing chlorimuron-ethyl, and verified by PCR.

### Deletion of Afcsp-1 in A. fumigatus

The Af*csp-1* (Afu1g10230) mutant was generated by gene replacement via double homologous recombination using the *pyrG* gene as a selectable marker^36^. Two primer pairs, Af10230_Pro_F (5′-GCGTCGACGCGGCCGCTGACAAACCCCTCTGGCGTAG-3′) and Af10230_Pro_R (5′-GGGAACGCGTGTGGCTGTTGCTCTGACTGAA-3′), and Af10230_Ter_F (5′-CGACGCGTCGATATCAAGGCAGACACGGGGAGA-3′) and Af10230_Ter_R (5′-CGGGATCCGATGCAGTGCTTAACCGACC-3′), were used to amplify the upstream and downstream flanking regions of Af*csp-1*, respectively. These two fragments were digested with SalI/MluI and MluI/BamHI, respectively, and then cloned into pBluescript II SK (Stratagene Co., La Jolla, CA, USA) to create construct pSK-UD. An 8.6-kb *pyrG* fragment was released by HpaI digestion of pCDA14, which was obtained from Cheng Jin’s laboratory, and then inserted into the site between the upstream and downstream regions of pSK-UD to yield the deletion construct pAfCSP-1-*pyrG*. After digestion with NotI, the linearized up-*pyrG*-down cassette was transformed into the WT strain CEA17 by a previously reported method^40^, and transformants were screened for uridine and uracil autotrophy. The transformants were confirmed by PCR using primers TestKO_F (5′-GGATCGGGCCTTGATGTTAC-3′) and TestKO_R (5′-GGACTTGCGCCGTCTACTGT-3′).

### ChIP-qPCR assay

The ChIP-qPCR assay was conducted in the WT and ∆wc*-2* strains (the negative control). Mycelial pieces (Φ = 1 cm) were inoculated into flasks containing 100 mL of Vogel’s liquid medium. The cultures were incubated at 28 °C with shaking at 180 rpm for 24 h in constant light, and then KTC was added to a final concentration of 2.5 μg/mL for 15, 30, and 60 min, respectively. The ChIP assay was performed as previously described^41^. The tissues were fixed in minimal medium containing 1% formaldehyde for 15 min at room temperature, and then glycine was added to a final concentration of 125 mM to stop the reaction. Chromatin was sheared into 500–1000-bp fragments by sonication. For each immunoprecipitation assay, 1 mL of protein (2 mg/mL) was used. The immunoprecipitation was performed using an anti-WC-2 antibody^42^. DNA was extracted and dissolved in 120 μL of distilled H_2_O. The DNA extraction was also conducted in no-antibody control samples to obtain the input DNA. Then, 2 μL of DNA solution was used for qPCR to quantitatively detect the *csp-1* promoter region bound by WC-2. The primers that cover the light regulatory elements were 5′-GTTGTGTGTATATCAGCGTAGG-3′ and 5′-CCCTTAGGTTCTTGGCAATC-3′. The qPCRs followed the protocol supplied in the SYBR Green Real-time PCR Master Mix (Toyobo, Osaka, Japan). qPCR detection was also conducted for the inputs. The ratio of the qPCR results between the ChIP sample and its input sample was calculated and presented as the % input to indicate WC-2 enrichment at the *csp-1* promoter. The experiment was independently performed three times.

### Sterol extraction and analysis

Strains were incubated at 28 °C with shaking at 180 rpm for 12 h in the dark, and then KTC was added to a final concentration of 2.5 μg/mL. After 24 h of incubation, mycelia were collected and dried for sterol extraction. Sterile extraction and analysis used previously reported methods^21^. The experiments were repeated twice.

## Additional Information

**How to cite this article**: Chen, X. *et al.* De-repression of CSP-1 activates adaptive responses to antifungal azoles. *Sci. Rep.* **6**, 19447; doi: 10.1038/srep19447 (2016).

## Supplementary Material

Supplementary Information

supplementary table S1

## Figures and Tables

**Table 1:** Strain list.

Strain	Genotype	Source
*N. crassa* FGSC #4200	Wild type	FGSC
*N. crassa* FGSC #11348	△*csp-1*; a	FGSC
*N. crassa* FGSC #11711	△*wc-1*; a	FGSC
*N. crassa* FGSC #11124	△*wc-2*; a	FGSC
*N. crassa* FGSC #13802	△*erg5*; a	FGSC
*N. crassa* FGSC #13803	△*erg5*; A	FGSC
*N. crassa* FGSC #18507	△*erg2*; a	FGSC
*N. crassa csp-1*^OE^	P*cfp::csp-1*	This study
*A. fumigatus* YJ-407	Wild type	Cheng Jin’s laboratory
*A. fumigatus* CEA17	△*pyrG*	Cheng Jin’s laboratory
*A. fumifatus* Af*csp-1*	△Afu1g10230	This study

**Table 2:** Gene-specific primer pairs used for qRT-PCR assay.

Gene	Forward primer sequence (5′ → 3′)	Reverse primer sequence (5′ → 3′)
β-tubulin	CCCAAGAACATGATGGCTGCTTCT	TTGTTCTGAACGTTGCGCATCTGG
NCU02713	ACTACAAGGACAAGTCGCGCTCAT	AAGGTTAAGTCCACGCATGTCCCA
NCU04156	TGAGCACCTTCACGATCTGTCCAA	TGATGTACATAGCACCCATGGCAC
NCU05278	TTTCACCTTCCTCTTCGCTTCCCA	TCATCGACTCAAGCTGCTCCATGT
NCU02624	AAATCGATTACGGCTACGGTCTCG	TATCGCTACCATCCACGTTCCTGA
NCU05591	GCTTTGGAAATGGATGGTGACGCT	AAATGCAGAGGGCGGTCTTAGAGT
NCU08899	AACTCTGCAGTGCCAAACCTCAAC	GGACGATGATGATGACTTGG
NCU04990	ACAGCTGCGAGACGACATACATGA	GGGATCGTTGTTGGGTAAG
