# Supplementary material for: De-repression of CSP-1 activates adaptive responses to antifungal azoles
Source: Sci Rep. 2016 Jan 19;6:19447. doi: 10.1038/srep19447 (PMC4726075; doi:10.1038/srep19447)
Supplement: Supplementary Information [file srep19447-s1.pdf]

Supplemental Information for

De-repression of CSP-1 activates adaptive responses to antifungal azoles

Xi Chen, Wei Xue, Jun Zhou, Zhenying Zhang,Shiping Wei, Xingyu Liu, Xianyun Sun, Wenzhao Wang, Shaojie Li*

Contents:

- Supplemental Figures S1 and S2

- Supplemental Table S2, and S3 (Supplemental Table S1 was attached in Excel)

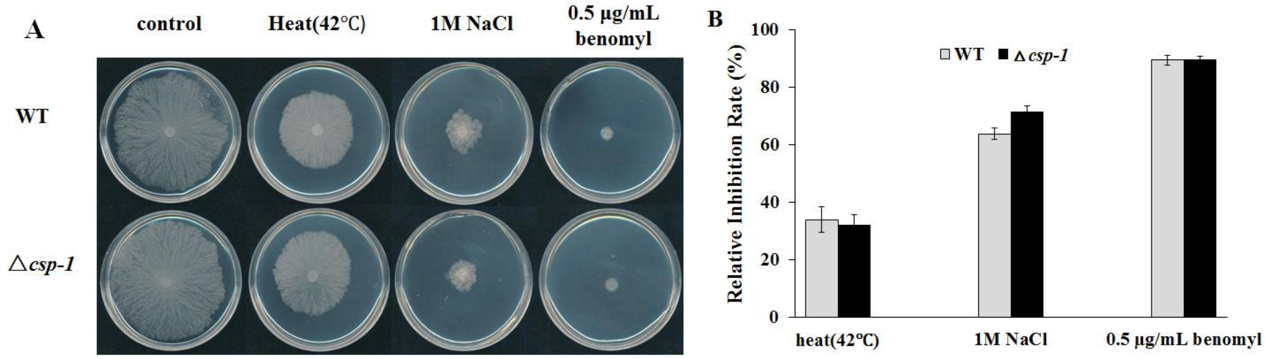

Figure S1 Effects of *csp-1* deletion in stress sensitivities. (A) Colony growth on plates with or without other stresses. Wild type (WT) and the *csp-1* deletion mutant (Δ*csp-1*) were inoculated under condition described in Materials and Methods. Mycelial plugs were incubated at 28 ℃ in dark. Images of colonies were captured after 24 h. (B) Relative growth inhibition rates by stresses. Relative growth inhibition rates were calculated based on diameters of colonies. Values of three replicates were used for statistic analysis. Means of inhibition rates were shown and standard deviations were marked with bars. p_{heat}=0.5879, n=3; p_{NaCl}=0.0145, n=3; $p_{benomyl}$=0.9576, n=3.

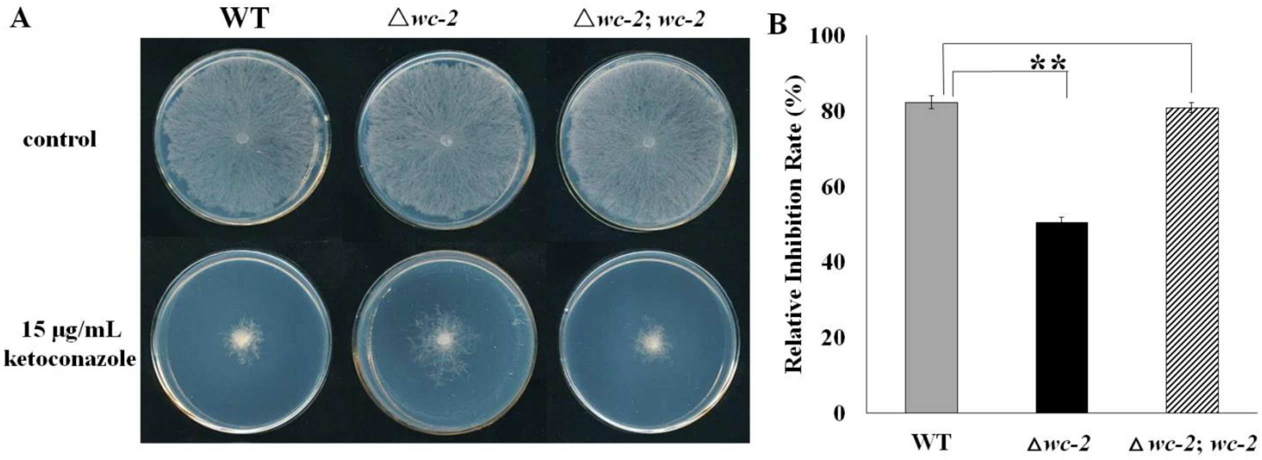

Figure S2 Effects of wc-2 deletion on azole sensitivity. (A) Azole sensitivity test. The mycelial plugs of WT, Δ*wc-2* and Δ*wc-2*; *wc-2* were inoculated on plates with or without azoles and incubated at 28 ℃ in dark. Images of colonies were captured after 24 h. (B) Relative growth inhibition rates. Relative growth inhibition rates were calculated based on diameters of colonies. Values of three replicates were used for statistic analysis. Means of inhibition rates were shown and standard deviations were marked with bars. $p_{\Delta wc\text{-}2}$=0.00002, n=3; $p_{\Delta wc\text{-}2;\, wc\text{-}2}$ =0.3091, n=3.

Table S2: Transcriptional responses by azole-responsive genes to *csp-1* deletion.

Gene	Control		Ketoconazole-treated	
	WT	∆*csp-1*	WT-K	∆*csp-1*-K
erg2	1.00±0.00	1.58±0.04**	4.68±0.31	5.67±0.09*
erg5	1.00±0.00	1.21±0.10	3.50±0.23	4.62±0.18**
erg11	1.00±0.00	1.33±0.28	12.82±0.61	18.48±0.62**
cdr4	1.00±0.00	1.16±0.12	8.57±0.19	16.09±0.30**
ads-1	1.00±0.00	1.70±0.13*	4.62±0.09	7.82±0.85*
stk-17	1.00±0.00	1.07±0.03*	1.99±0.13	4.63±0.52**

Note: WT: wild-type strain; ∆*csp-1*: *csp-1* deletion strain; Transcript levels were calculated elative to those for the wild-type strain withou KTC treatment. Significances between mutant and WT were estimated by the T-test, strains with extreme significant difference (P<0.01) were marked with **, strain with significant difference (0.01<P<0.05) were marked with *, strains without significant differences were not marked.

Table S3: Transcriptional responses by azole-responsive genes to *csp-1* overexpression.

Gene	Control		Ketoconazole-treated	
	WT	*csp-1*^{OE}	WT-K	*csp-1*^{OE}-K
erg2	1.00±0.00	0.61±0.01**	3.99±0.11	1.41±0.09**
erg5	1.00±0.00	1.25±0.16	5.04±0.46	3.19±0.20*
erg11	1.00±0.00	1.15±0.06*	13.39±0.22	9.46±0.75**
cdr4	1.00±0.00	1.25±0.15	15.90±2.20	4.21±0.16*
ads-1	1.00±0.00	1.55±0.15*	11.80±0.94	4.14±0.13**
stk-17	1.00±0.00	1.04±0.06	5.35±015	0.85±0.05**

Note: WT: wild-type strain; *csp-1*^{OE}: *csp-1* overexpression strain; Transcript levels were calculated relative to those for the wild-type strain withou KTC treatment. Significances between *csp-1*^{OE} and WT were estimated by the T-test, strains with extreme significant difference (P<0.01) were marked with **, strain with significant difference (0.01<P<0.05) were marked with *, strains without significant differences were not marked.
